# Exploring the Application of Pattern Recognition and Machine Learning for Identifying Movement Phenotypes During Deep Squat and Hurdle Step Movements

**DOI:** 10.3389/fbioe.2020.00364

**Published:** 2020-04-29

**Authors:** Sarah M. Remedios, Daniel P. Armstrong, Ryan B. Graham, Steven L. Fischer

**Affiliations:** ^1^Occupational Biomechanics and Ergonomics Laboratory, Department of Kinesiology, University of Waterloo, Waterloo, ON, Canada; ^2^Spine Biomechanics Laboratory, School of Human Kinetics, University of Ottawa, Ottawa, ON, Canada

**Keywords:** principal component analysis, cluster, gaussian mixture model, movement phenotypes, functional movement screen

## Abstract

**Background:**

Movement screens are increasingly used in sport and rehabilitation to evaluate movement competency. However, common screens are often evaluated using subjective visual detection of *a priori* prescribed discrete movement features (e.g., spine angle at maximum squat depth) and may not account for whole-body movement coordination, or associations between different discrete features.

**Objective:**

To apply pattern recognition and machine learning techniques to identify whole-body movement pattern phenotypes during the performance of exemplar functional movement screening tasks; the deep squat and hurdle step. Additionally, we also aimed to compare how discrete kinematic measures, commonly used to score movement competency, differed between emergent groups identified via pattern recognition and machine learning.

**Methods:**

Principal component analysis (PCA) was applied to 3-dimensional (3D) trajectory data from participant’s deep squat (DS) and hurdle step performance, identifying emerging features that describe orthogonal modes of inter-trial variance in the data. A gaussian mixture model (GMM) was fit and used to cluster the principal component scores as an unsupervised machine learning approach to identify emergent movement phenotypes. Between group features were analyzed using a one-way ANOVA to determine if the objective classifications were significantly different from one another.

**Results:**

Three clusters (i.e., phenotypes) emerged for the DS and right hurdle step (RHS) and 4 phenotypes emerged for the left hurdle step (LHS). Selected discrete points commonly used to score DS and hurdle step movements were different between emergent groups. In regard to the select discrete kinematic measures, 4 out of 5, 7 out of 7 and 4 out of 7, demonstrated a main effect (*p* < 0.05) between phenotypes for the DS, RHS, and LHS respectively.

**Conclusion:**

Findings support that whole-body movement analysis, pattern recognition and machine learning techniques can objectively identify movement behavior phenotypes without the need to *a priori* prescribe movement features. However, we also highlight important considerations that can influence outcomes when using machine learning for this purpose.

## Introduction

Movement screens are commonly used to assess an individual’s quality of movement as a method to highlight poor movement patterns (McCunn et al., 2016). The quality of movement, herein termed movement competency, can be explained as an individual’s ability to adopt a movement pattern that achieves the task objective, while also minimizing injury risk (Kritz et al., 2009; McGill et al., 2015). Considering the emphasis on movement and safety, sport (McCunn et al., 2016) and occupational settings (Isernhagen, 1992; Sinden et al., 2017) rely on movement screening methods to estimate performance capacity (Frost et al., 2015; Bock et al., 2016) and to reveal functional limitations that may increase risk of injury (O’Connor et al., 2011; Lisman et al., 2013). Visual assessment of body mechanics is the *de facto* method for measuring movement competency (Sinden et al., 2017), which increases the subjectivity of movement screens, thus relying on the appraisal and previous knowledge of the practitioner. As a result, it may not be surprising that inter-rater reliability issues continue to restrict the utility of movement screening approaches (i.e., Shultz et al., 2013).

In addition to inter-rater reliability challenges that affect subjective appraisal of movement competency, the current use of top-down, prescribed, discrete movement features to define “safe” or “good” movement may be inadequate. While many believe that movement competency is linked to injury risk and/or performance (where movement competency is defined using conventional *a priori* definition such as torso is parallel with the tibia when performing the deep squat), there remains little evidence supporting such connections (Gross and Battié, 2006; Mottram and Comerford, 2008; Schneiders et al., 2011; Okada et al., 2011; Parchmann and McBride, 2011). Perhaps our *a priori* criteria for subjectively evaluating movement competency are incorrect or incomplete (Bennett et al., 2017), or our clinical eye is simply not appropriately tuned to detect important and meaningful changes. As an alternative to this top-down approach, use of emerging tools in machine learning might help us to identify naturally-occurring movement phenotypes, where continued research can then explore phenotypes that are associated with positive or negative health outcomes with respect to specific task objectives.

Considering the magnitude of variability that exists in the ways individuals can complete a task, a reliance on discrete *a priori* measures, as common movement screen scoring parameters (e.g., spine angle at maximum squat depth), instead of assessing time-series whole-body movement patterns remains as a limitation. Specifically, the use of *a priori* discrete parameters suggests that there is a single idealized pattern, which as shown by Srinivasan and Mathiassen (2012), is not necessarily optimal. Instead, it may be more beneficial to identify and screen for phenotypical patterns of movement behaviors that may differentiate and classify between those with optimal movement competency relative to those that may benefit from a targeted movement training intervention.

The Functional Movement Screen^TM^ (FMS) (Functional Movement Systems, Chatham, VA, United States) remains a popular tool for movement screening (Bennett et al., 2017). Sinden et al. (2017) identified the FMS^TM^ as one of the most commonly used approaches for movement screening among Kinesioligists. The FMS^TM^ is an example of a movement screen that depends on the visual appraisal of discrete movement competency and identifies deficits and/or compensatory movement patterns in the kinetic chain (Cook et al., 2006a, b). While the FMS^TM^ protocol includes a battery of 7 distinct movements, we focus on the Deep Squat (DS) and right and lift hurdle step (RHS; LHS) movements. Squatting is a common pattern in most athletic events (Cook et al., 2006a; Kritz et al., 2009) making it a useful movement to target first. The hurdle step movement, provides a unique contrast relative to the squat because it tests bilateral functional mobility and dynamic stability of the hips, knees, and ankles (Cook et al., 2006a). Many believe that such screening can be useful in proactive injury prevention (Kiesel et al., 2007). However, due to the lack of evidence relating the FMS^TM^ to injury (McCunn et al., 2016), or biomechanical exposure variables in transfer tasks (Beach et al., 2014), evidence does not support that the current scoring approach is useful for injury prevention (Okada et al., 2011; Parchmann and McBride, 2011). This is not, however, to suggest that screening is not useful. Considering sound biomechanical arguments (Zazulak et al., 2008; Powers, 2010; Hewett and Myer, 2011), Beach et al. (2014), conclude that general whole-body movement screening could be used to predict likelihood of injury in physically demanding jobs if we advance beyond the current scoring approaches. Therefore, to overcome limitations associated with the subjective *a priori* driven grading criteria, data-driven methods could improve the state of movement screening (McCunn et al., 2016).

Application of pattern recognition and machine learning techniques are growing within biomechanics (Halilaj et al., 2018) and can enable data-driven methods to objectively identify movement phenotypes. As a pattern recognition tool, principal component analysis (PCA), allows us to identify principal movement patterns through data reduction, which explain variance within kinematic-based data sets (Troje, 2002; Wrigley et al., 2005; Brandon et al., 2013; Federolf et al., 2014; Ross et al., 2018; Armstrong et al., 2019). One strength of using PCA to determine modes of variability is that the scores can be used in downstream analysis such as in classification through cluster analysis to detect and interpret differences between subjects and/or trials (Deluzio et al., 2014, p. 319). As an example, clustering is an unsupervised machine learning method that iteratively clusters data points into groups assigning each observation to a cluster. In the biomechanical analysis of human movement data, clustering has proven useful for grouping participants with similar patterns (Sawacha et al., 2010; Bennetts et al., 2013; Gilles and Wild, 2018) and gait waveforms (Watelain et al., 2000; Toro et al., 2007; Roche et al., 2014). Previously, PCA and clustering techniques have been combined to identify and group distinct spine spatiotemporal movement strategies (Beaudette et al., 2019), which support that a combination of these methods may have utility in objectively identifying movement phenotypes in a movement screening context. However, such application of pattern recognition and clustering to identify naturally occurring movement phenotypes within the movement screening context remains a novel endeavor.

Therefore, to address issues related to the use of subjectively measured *a priori* movement competency features, the objective of this paper was to apply PCA and gaussian mixture model (GMM), as pattern recognition and machine learning techniques respectively, to objectively identify naturally occurring whole-body movement pattern phenotypes during the performance of common movement screening tasks (i.e., the deep squat and hurdle step). Secondarily, we aimed to evaluate if top-down *a priori* determined, discrete kinematic variables (typically evaluated in practice using a subjective visual appraisal), were indeed different between naturally emerging movement phenotype groups identified using unsupervised learning (i.e., bottom-up).

## Materials and Methods

### Subjects

Thirty healthy participants volunteered for this study (Table 1). The participants were recruited from the general student body of the University of Waterloo, were older than 18 years old and did not have an injury that prevented activities of daily living in the previous 6 months. The participants completed a “Get Active Questionnaire” that indicated their physical readiness for the study. This study was approved by the University of Waterloo’s Office of Research Ethics, and participants provided informed consent prior to participation.

**TABLE 1:** Participant demographics.

	Age	Height (cm)	Weight (kg)
Male (*n* = 15)	23.6 ± 4.0	185.23 ± 6.8	87.9 ± 10.0
Female (*n* = 15)	23.7 ± 8.0	168.2 ± 9.8	64.3 ± 9.25

### Instrumentation

**FIGURE 1:**
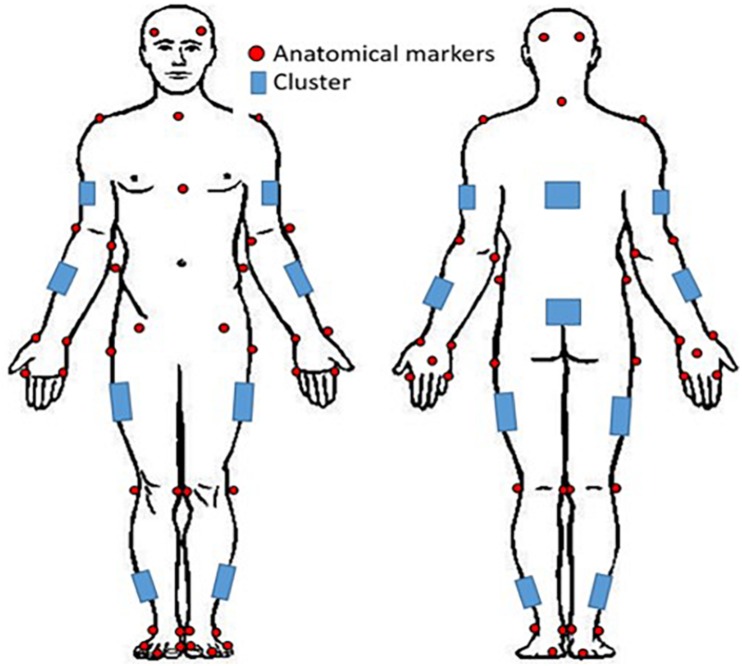
Whole-body reflective marker set up. Marker clusters were placed bilaterally on the shank, thigh, forearm, and upper arm as well as on the pelvis and trunk. Anatomical markers were placed on the anterior and posterior lateral aspects of the head, suprasternal notch, xiphoid process, 7^th^ cervical vertebra; and bilaterally on the acromion, lateral and medial epicondyles, radial and ulnar styloid processes, 2nd and 5th metacarpals, dorsum of the hand, iliac crest, anterior superior iliac spine, greater trochanter of femur, lateral and medial condyles, lateral and medial malleoli, 1st and 5th metatarsal, dorsal tarsal midline, and calcaneus.

Prior to coming to the lab, participants were instructed to wear tight fitting clothing. All participants were instrumented with reflective motion capture markers, including marker clusters placed over body segments and single markers positioned over anatomical landmarks (Figure 1). Marker clusters were used to track segment motion instead of anatomical markers to reduce soft tissue artifact (Leardini et al., 2005). Anatomical markers were used during calibration to mathematically relate the technical coordinate system of each cluster to its underlying segment specific anatomical coordinate system (Robertson et al., 2013). Motion was recorded using a 12 – camera (six, Vantage v5; six, Vero v2.2) Vicon Nexus 2.6 motion capture system (Nexus, Oxford, United Kingdom). Once participants completed a calibration trial, the following markers, bilaterally, were removed for the remainder of the study: lateral and medial epicondyles, iliac crest, anterior superior iliac spine, greater trochanter of femur, lateral and medial condyle, lateral and medial malleolus.

### Protocol

**FIGURE 2:**
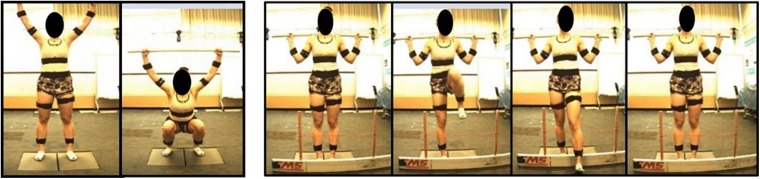
Deep squat movement and hurdle step movement as adapted from Cook et al. (2006a).

Participants performed 15 movements in total for the study: 5 deep squat (DS), 5 right hurdle step (RHS), and 5 left hurdle step (LHS) movements (Figure 2) and were given instruction about how to perform each movement, adapted from those described in Cook et al. (2006a). Specifically, for the deep squat participants were instructed to: “stand with your feet approximately shoulder width apart, place the dowel on your head adjusting your hands until your elbows are at 90 degrees, press the dowel overhead, straightening the elbows, the trial will begin once you descend into a deep squat position and back up keeping your heels on the floor and arms extended the entire time.” For the hurdle step, instructions were: “stand facing the front of the lab with your toes touching the FMS board, place the dowel across the back of your shoulders and below your neck, with your right/left leg, step over the hurdle, touch your heel on the opposite side and bring your moving leg back to the starting position.” Participants completed 5 repetitions of the DS, followed by 5 repetitions of the RHS and 5 of the LHS. Motion data were collected at 60 Hz using Vicon Nexus while participants performed the DS, RHS and LHS movements, respectively.

### Data Post-processing and Conditioning

During post-processing, each trial was first labeled and gap filled in Vicon Nexus, where gaps were filled using cubic spline, pattern fill or rigid body fill functions within Vicon Nexus, where the gap filling function was dependent on the underlying gap length (Armstrong et al., 2019). Gap-filled and labeled marker trajectory data were exported to Visual 3D (C-Motion Inc., Germantown, United States). Within Visual 3D, data were filtered using a fourth order low pass butterworth filter with an effective cut off frequency of 6 Hz (Winter, 2009) to remove high frequency noise from each signal. Filtered trajectory data were then used to drive a 15 segment whole-body kinematic model, with IK constraints, where segments were defined using ISB recommended segment definitions (Wu et al., 2002, 2005), such that joint center positions (ankle, knee, hip, shoulder, elbow, wrist) and centre of mass (COM) locations (pelvis, trunk, and head) could be calculated. Joint center and COM trajectory data were combined with filtered position data from selected body landmarks (xiphoid process, suprasternal notch, 7th cervical vertebra) to provide the kinematic description of each motion.

**FIGURE 3:**
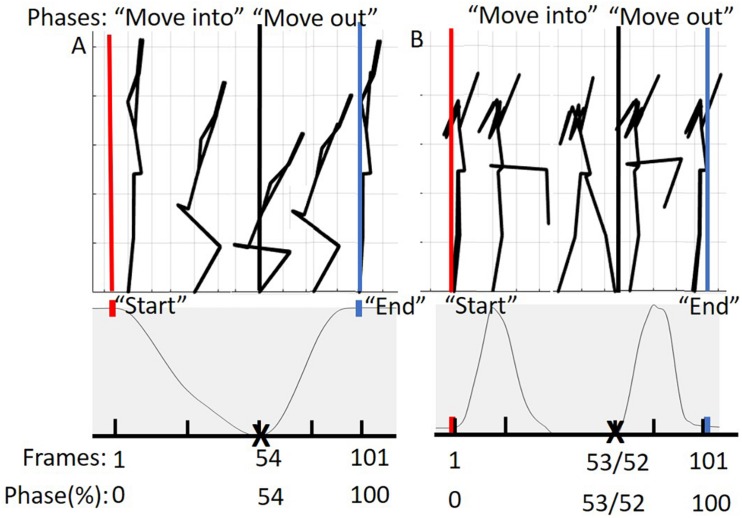
**(A)** Represents the phases of the DS movement from 0 to 100%. The “move into” phase is contained by the red and black bars and in between the black and blue bars is the “move out” phase. The below graph represents tracking of the suprasternal notch marker to determine 50% of the movement (maximum squat depth) as well as define “start” and “end” points of the movement. **(B)** Represents the phases of the hurdle step movement from 0 to 100%. The “move into” phase is contained by the red and black bars and in between the black and blue bars is the “move out” phase. The graph below represents tracking of the right (LHS) or left (RHS) calcaneus marker to determine 50% of the movement (end of heel touch) as well as define “start” and “end” points of the RHS and LHS, respectively.

Prior to additional data processing, start and end frames for each trial were determined (Figure 3). The DS “start” and “end” were defined by identifying the local maximum of the supra-sternal notch marker in the vertical direction. The “start” and “end” of the hurdle step were determined by identifying the local minimum of the lead (step-over) heel marker in the vertical direction.

To address our overarching research question, we also used the trajectory data to calculate discrete kinematic variables commonly used in screening. Tables 2, 3 list the additional discrete kinematic data that were calculated and also summarizes how they were calculated to support this analysis.

**TABLE 2:** Calculated kinematic variables typically representative of the *a priori*, discrete DS scoring criteria.

Scoring criteria (Cook et al., 2006a)	Kinematic representation	Calculation	Meaning/Relevance
The femur is at or below horizontal	Femoral angle	The right and left femoral segment angles were calculated, averaged, and then the maximum angle was found.	Representation of squat depth
The trunk is parallel with the tibia and/or toward the vertical	Trunk- Shank angle difference Trunk angle	The maximum trunk to shank angle difference was determined as well as the maximum trunk angle elicited.	The greater the difference the more the tibia and trunk are not parallel. The greater the trunk angle, the further bent forward.
The knees remain aligned with the feet	Knee-to-ankle separation ratio (Ortiz et al., 2016)	The difference between left and right knee joint centers in global space and the difference between the left and right ankle joint centers in global space were calculated. The knee distance was divided by the ankle distance.	(>1) = knees are wider (varus) (<1) = knees fall inwards (valgus)
The dowel remains over top of the feet	Left-right hand center difference to foot center difference.	The center of the left- and right-hands in global space and left and right foot center in global space were calculated. The maximum anterior difference between the hand center and foot center was calculated.	Measure of displacement of dowel over the feet in the anterior direction.

**TABLE 3:** Calculated kinematic variables typically representative of the *a priori*, discrete hurdle step scoring criteria.

Scoring criteria (Cook et al., 2006a)	Kinematic representation	Calculation	Explanation
Hips, knees, and ankles remain aligned	Hip-to-Knee difference Knee-to-ankle difference Hip-to-ankle difference Hurdling leg was the leg of interest	=(hip – knee) = (knee – ankle) = (hip-ankle) All calculations used the joint center in the y-axis (anterior plane) Peak absolute values were calculated	A difference value closer to 0, the more in-line the joint centers. The hurdle step scoring criteria #1, was determined kinematically by gathering the difference between all three joints in the anterior plane.
Little to no movement noted in lumbar spine	Lumbar Flexion – extension range Lumbar lateral flexion range Rotation range	=(maximum extension – maximum flexion) = (maximum right lateral flexion – maximum left lateral flexion) = (maximum rotation to the right – maximum rotation to the left)	To determine little movement in lumbar spine, angle ranges in all three directions were calculated. The greater the range, the more movement noted in lumbar spine.
The dowel remains parallel with the string	Right-left hand difference in the z direction (superior/inferior)	=(right hand center – left hand center)	Represented by the difference in hand displacement in the superior/inferior direction. The greater the difference of the two hands, the greater the dowel is not parallel with the string.

To support the use of pattern recognition and machine learning, trajectory data representing the above mentioned joint centers, landmarks, and COM locations were exported to Matlab (MathWorks, Natick, United States). In Matlab, participants’ trajectory data were divided by their standing height to normalize for inter-participant variance in height (Ross et al., 2018). The trajectory data were also translated such that the new origin was positioned at the center of the right (DS, LHS) or left (RHS) ankle coordinate system. This translation was necessary to eliminate variance in the trajectory data associated with each participants’ relative positioning with the global coordinate system of the laboratory.

Trials were time normalized to 101 frames (100% of the trial), but in phases in order to account for the fact that participants typically took longer to move into the required position, but less time to move out of the required position. As an example, participants for the DS generally took different lengths of time descending into maximum squat depth and returning to upright standing. To achieve our desired phase-based time-warping, first, the “move into” portion of the movement (i.e., from standing to maximum squat depth, or foot extended over hurdle) was segmented out and time normalized to 54 frames for the DS, 53 for the RHS and 52 for the LHS, respectively. Second, the “move out” portion of the movement (i.e., return to standing) was segmented out and time normalized to 47 frames for the DS, 48 for the RHS and 49 for the LHS respectively. Third, the time normalized phases were re-concatenated into a complete trial (101 frames). On average, participants tended to complete the “move into” phase of the DS, RHS, and LHS at 54, 53, and 52% of the total movement time, reinforcing the splits noted above. This process was completed to eliminate timing effects or phase shift between trials of each of the movements respectively (Moudy et al., 2018). The time normalized estimated joint centers, body landmarks, and calculated COM positions were then prepped for PCA analysis in Matlab (Figure 3).

### Data Analysis

#### Feature Selection

PCA was applied to the time-series conditioned and post-processed trajectory data to identify emergent features that captured orthogonal modes of variability in the data set. Individual PCA models were developed for the DS, RHS, and LHS data, respectively, using the ‘Statistics and Machine Learning’ toolbox in Matlab. Described more completely in Ross et al. (2018), but briefly summarized here, we organized the time-series trajectory data into a [n, m] matrix, where, n represented the number of trials (*n* = 150, corresponding to 30 participants × 5 trials) and where m represented row vectors describing the time-series trajectory data (*m* = 5454, corresponding to 18 trajectories × 3 axes × 101 time points). PCA was then applied as a data reduction and feature selection method to yield principal components (PCs) that capture linearly uncorrelated sources of variability within each dataset. The application of PCA in this manner, for the purpose of identifying principal movements (PCs representing linearly uncorrelated movement features) is more completely described by Troje (2002) and Ross et al. (2018). PC scores were retained, representing each observation (trial) in the principal component space. PCs that individually explained > 5% of the variance (Witte et al., 2010), were retained for classification.

#### Classification

A [p, q] matrix was input into a GMM, where p represents each trial’s PC scores as input features (*p* = 150, corresponding to 30 participants × 5 trials) and each column of q described individuals’ PC scores for those PCs that were retained. As a brief background, GMM is a model-based method where the algorithm is aimed at optimizing the fit between the data and the model to find structures (clusters) among the observations, while also assigning a measure of probability to the clustered assignment. GMM was applied to the data in Matlab using the “Statistics and Machine Learning” toolbox. To determine the optimal *k* (number of clusters), we used the Bayesian information criterion (BIC), where for *k* = 1–10 a GMM was fit to the dataset and the minimum BIC identified the best *k.* An optimal *k* was determined for each movement: DS, RHS and LHS, respectively. A GMM for each movement was applied to each data set respectively, running 100 repetitions to increase the likelihood of the data converging to an optimum (Beaudette et al., 2019). Following the application of the GMM to each movement dataset, centroid scores from each cluster were determined along with the clustering assignments from each individual trial, where hard clustering was performed such that each trial was assigned to only 1 phenotype. The cluster centroids therefore represent the mean movement phenotypes.

#### Reconstruction

Single component reconstruction was used to visualize differences in movement patterns between clusters (Brandon et al., 2013). This reconstruction was done by multiplying the loading vectors for each retained PC by the centroid scores representing each cluster and adding it to the mean loading vector (eigenvectors from the PCA models). The reconstructed patterns provided a visual representation that emphasizes differences in the underlying kinematics associated with each movement phenotype.

#### Statistics

Kinematic variables typically representative of the *a priori*, discrete scoring criteria (Tables 2, 3) served as dependent variables in one-way ANOVA models. Cluster assignment served as the independent variable (3 levels for DS and RHS movements and 4 for LHS movement, based on the emergence of 3 and 4 clusters, respectively). An alpha value of 0.05 was used to determine significance. Where a main effect of cluster assignment emerged, *post hoc* testing, using Bonferonni corrected pairwise comparisons were used to determine significant differences in dependent measures between clusters. Partial eta squared values (η^2^) were calculated for each dependent variable where, 0.01 was considered a small effect, 0.06 a medium effect and 0.14 a large effect (Cohen, 1988, p. 285–287, 383). Statistical analysis was completed using SPSS (SPSS Version 24.0, IBM Corporations, Armonk, NY, United States).

## Results

### Feature Selection and Classification

**FIGURE 4:**
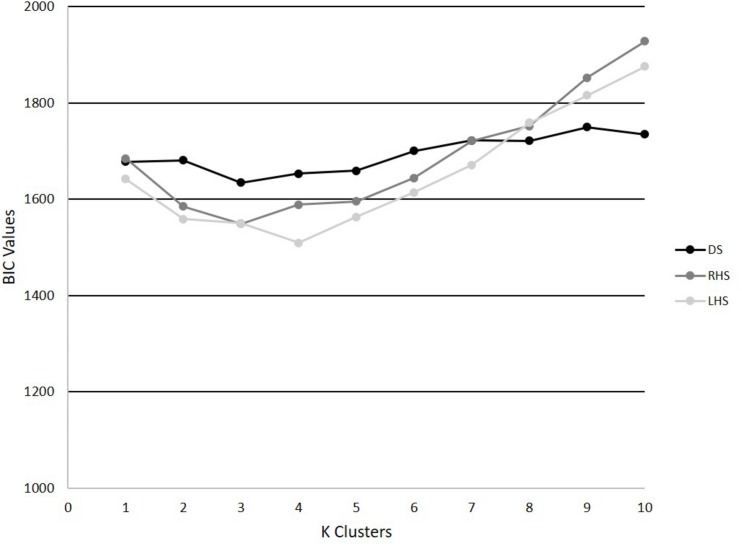
BIC values for *k* = 1–10 for the DS, RHS, and LHS demonstrating minimum values at *k* = 3 for the DS and RHS, and *k* = 4 for the LHS.

The PCA models revealed that 4, 6, and 6 PCs each explained at least 5% of the variance in the time-series trajectory data for the DS, RHS, and LHS, respectively. Using those retained PCs, the GMM identified *k* = 3 as the optimal number for the DS and RHS and *k* = 4 for the LHS movements (Figure 4). For the DS, 62, 24, and 64 trials were assigned to phenotypes 1, 2, and 3, respectively, where 23 of the participants had all 5 trials classified within the same phenotype and 7 participants had trials distributed between 2 different phenotypes. For the RHS, 47, 84, and 19 trials were assigned to phenotypes 1, 2, and 3, respectively, where 21 of the participants had all 5 trials classified into the same phenotype, and 9 participants had trials distributed between different phenotypes. Lastly, for the LHS, 36, 50, 25, and 39 trials were assigned in phenotypes 1, 2, 3, and 4 respectively, where 23 participants had all 5 trials classified within the same phenotype, and 7 participants had trials distributed between different phenotypes. It is interesting to note the disproportionate clustering, where many trials were assigned to cluster 2 for the RHS, but fewer to clusters 1 or 3, as an example. It is important to note than when interpreting the data, each movement was analyzed separately, for example, we cannot claim that phenotype 1 for the RHS and phenotype 1 for the LHS are related.

### Single Component Reconstruction

**FIGURE 5:**
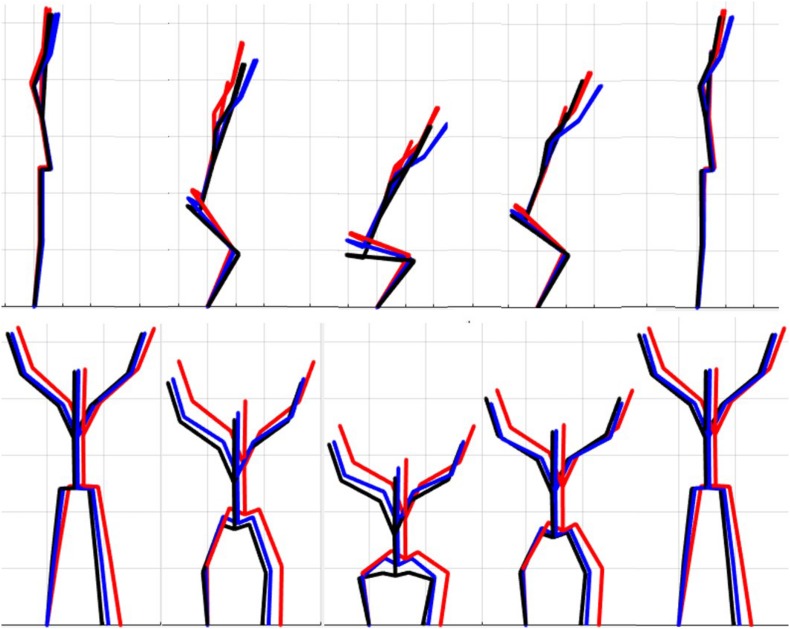
Reconstructed movement phenotypes using the centroid PC scores from each cluster considering the deep squat movement. Black, movement phenotype 1; red, movement phenotype 2; blue, movement phenotype 3.

**FIGURE 6:**
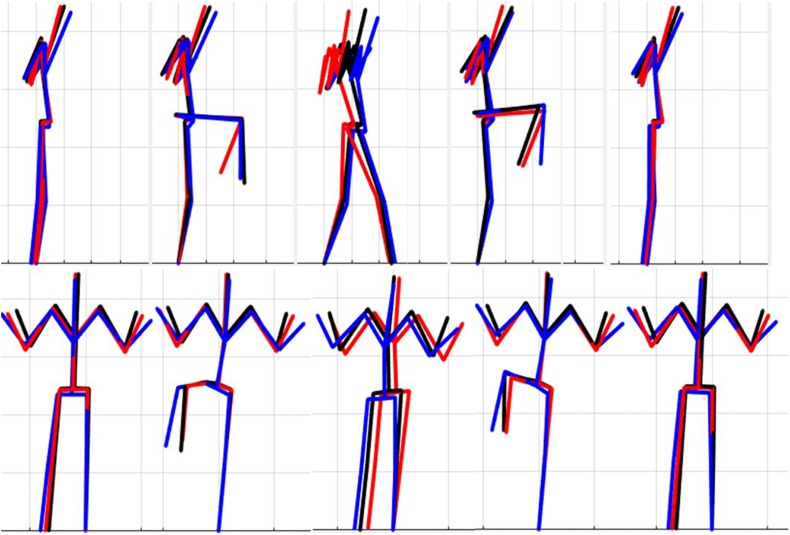
Reconstructed movement phenotypes using the centroid PC scores from each cluster considering the right hurdle step movement phenotypes identified. Black, movement phenotype 1; red, movement phenotype 2; blue, movement phenotype 3.

**FIGURE 7:**
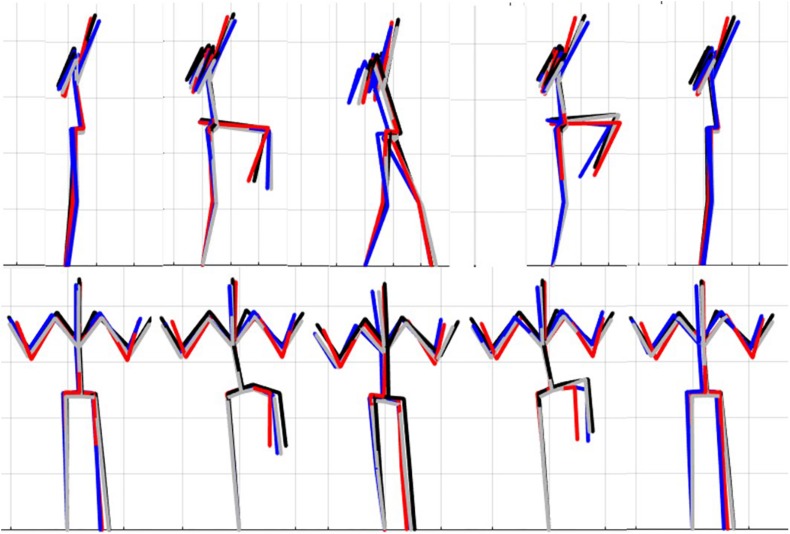
Reconstructed movement phenotypes using the centroid PC scores from each cluster considering the left hurdle step movement phenotypes identified. Black, movement phenotype 1; red, movement phenotype 2; blue, movement phenotype 3; gray, movement phenotype 4.

The results of the single component reconstructions are in Figures 5–7 and in Supplementary Material. The purpose of the single component reconstructions is to provide a visual representation of the emergent differences in movement phenotypes (cluster centroids).

### Kinematic Differences Between Clusters

#### Deep Squat

**FIGURE 8:**
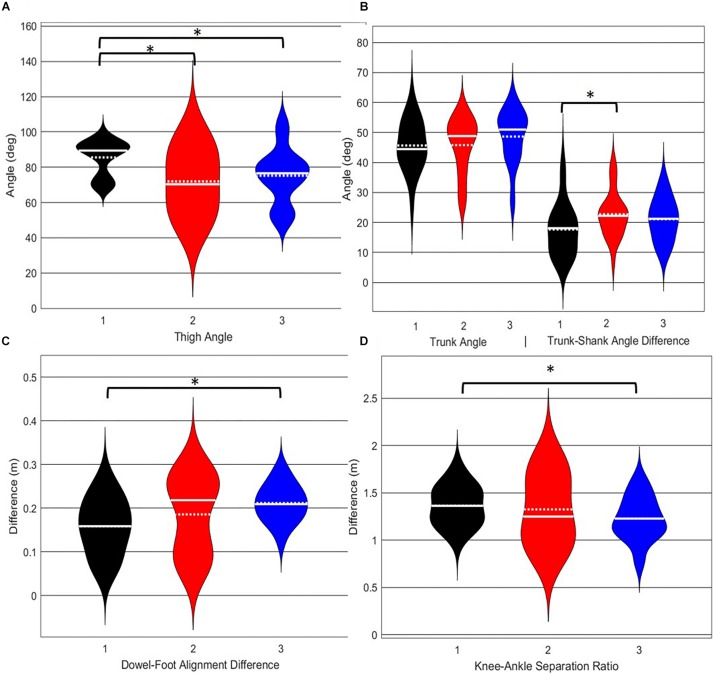
Violin plot (Holger Hoffmann, 2020) demonstrating the distribution shape of each phenotype for kinematic measures commonly used to score the DS. The mean is represented by the white dotted line and median with the solid white line. **(A)** The femur is at or below horizontal; **(B)** the torso remains upright and/or is parallel with the tibia; **(C)** the dowel remains aligned over the feet; **(D)** the knees remain aligned with the feet. *The mean difference is significant at the 0.05 level.

A main effect of cluster assignment was detected for 4 of the 5 kinematic measures typically used to subjectively evaluate the DS (Table 4 and Figure 8). The trunk segment angle, a measure to represent forward lean of the torso, was not different between the clusters. *Post hoc* pairwise comparisons revealed that the femoral angle (Figure 8A), was different between phenotypes 1 and 2 and phenotypes 1 and 3, but that phenotypes 2 and 3 were not different. Considering the trunk-shank angle difference measure (Figure 8B) phenotypes 1 and 2 were different, but phenotype 3 was not different from either 1 or 2. The sagittal plane dowel alignment, was different between phenotypes 1 and 3, but phenotype 2 was not different from either 1 or 3 (Figure 8C). Lastly, the knee-ankle separation ratio, a measure aimed to represent knee varus/valgus, showed differences between phenotypes 1 and 3 only (Figure 8D).

**TABLE 4:** Level of significance (*p*-value) and effect size (η^2 =^ partial eta squared value) results for the kinematic variable representation of the DS scoring criteria.

	The thigh is at or below horizontal	The trunk remains upright and/or remains parallel with the tibia		The Dowel remains aligned over feet	The knees remain aligned with the ankle
				
	Thigh segment angle	Trunk segment angle	Trunk shank angle difference	Hand center – foot center difference	Knee ankle separation ratio
Deep squat	*p* = 0.000* η^2^ = 0.141	*p* = 0.140 η^2^ = 0.026	*p* = 0.011* η^2^ = 0.059	*p* = 0.000* η^2^ = 0.143	*p* = 0.009* η^2^ = 0.063

*Denotes a main effect at the 0.05 level.

#### Right Hurdle Step

**FIGURE 9:**
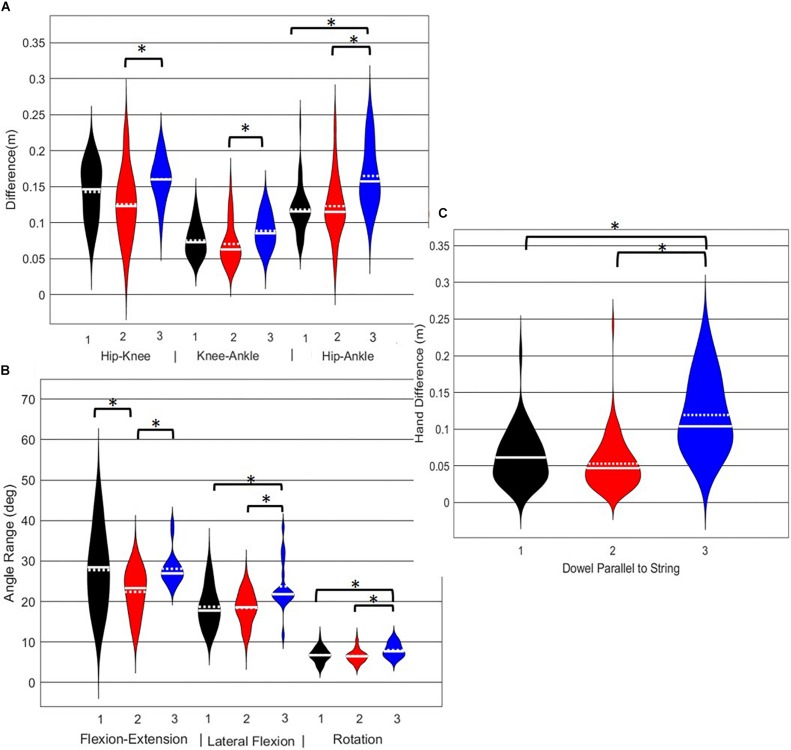
Violin plot (Holger Hoffmann, 2020) demonstrating the distribution shape of each phenotype for kinematic measures commonly used to score the RHS. The mean is represented by the white dotted line and median with the solid white line. **(A)** Hip, knee, and ankle remain aligned; **(B)** there is little to no movement in the lumbar spine; **(C)** the hands/dowel remains parallel to the string. ^∗^The mean difference is significant at the 0.05 level.

A main effect of cluster assignment was detected for all 7 kinematic measures commonly used to score the RHS, implying these variables soundly represent variance in the movement of the RHS (Table 5). *Post hoc* pairwise comparisons revealed that all three hip-knee-ankle frontal plane alignment variables were significant between phenotypes 2 and 3, and where the ankle-hip alignment variable was different between phenotypes 1 and 3 (Figure 9A). Considering measures associated with lumbar spine control, *post hoc* pairwise comparisons revealed differences between phenotypes 2 and 3 in the range of motion about all three axes (Figure 9B). There were further differences between phenotypes 1 and 3 for both the lumbar range of motion associated with a lateral bend and rotation. Phenotypes 1 and 2 only differed for the range of lumbar movement represented by the flexion/extension axis. Lastly, consider the hands/dowel parallel to the string measure, there were significant difference between phenotypes 1 and 3, and 2 and 3 (Figure 9C).

**TABLE 5:** Level of significance (*p*-value) and effect size (η^2^ = partial eta squared value) results for the kinematic variable representation of the hurdle step scoring criteria.

	The hips, knees, ankle remain aligned			There is little to no movement in the lumbar spine			Dowel parallel with string
	Hip-knee	Knee-ankle	Hip-ankle	Flex/Ext range	Lat flex range	Rotation range	Hands parallel
Right hurdle step	*p* = 0.003*	*p* = 0.026*	*p* = 0.000*	*p* = 0.000*	*p* = 0.000*	*p* = 0.004*	*p* = 0.000*
	η^2^ = 0.077	η^2^ = 0.048	η^2^ = 0.120	η^2^ = 0.139	η^2^ = 0.127	η^2^ = 0.074	η^2^ = 0.258
Left hurdle step	*p* = 0.238	*p* = 0.418	*p* = 0.008*	*p* = 0.001*	*p* = 0.000*	*p* = 0.932	*p* = 0.000*
	η^2^ = 0.028	η^2^ = 0.019	η^2^ = 0.078	η^2^ = 0.112	η^2^ = 0.225	η^2^ = 0.003	η^2^ = 0.284

*Denotes a main effect at the 0.05 level.

#### Left Hurdle Step

**FIGURE 10:**
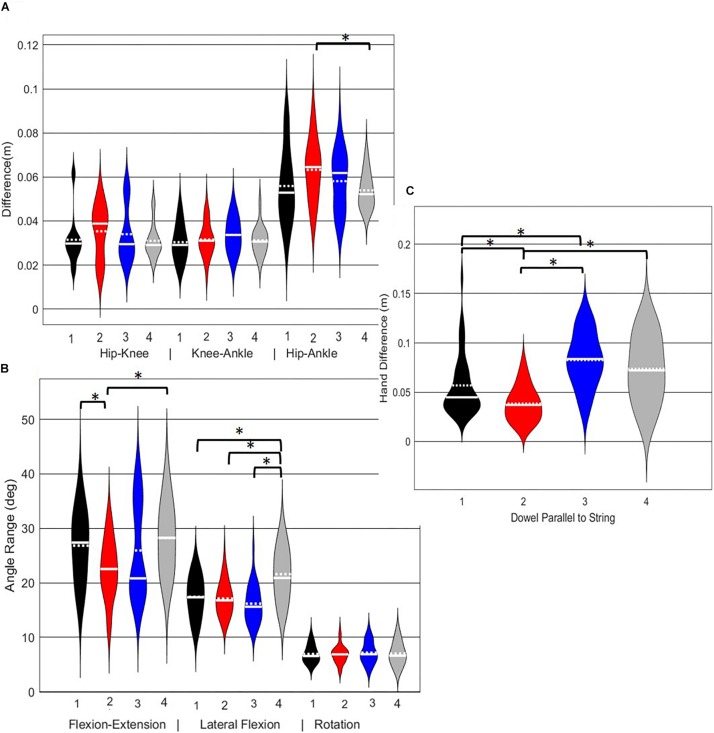
Violin plot (Holger Hoffmann, 2020) demonstrating the distribution shape of each phenotype for kinematic measures commonly used to score the LHS. The mean is represented by the white dotted line and median with the solid white line. **(A)** Hip, knee, and ankle remain aligned; **(B)** there is little to no movement in the lumbar spine. **(C)** The hands/dowel remains parallel to the string. ^∗^The mean difference is significant at the 0.05 level.

A main effect of cluster assignment was detected for 4 of the 7 kinematic measures commonly used to score LHS, including hip-ankle alignment, lumbar flexion/extension range, lumbar lateral flexion range, and hands/dowel parallel with the string measure. No main effects were detected for hip-knee and knee-ankle alignment difference and lumbar rotation range (Table 5). *Post hoc* pairwise comparisons revealed that hip-ankle alignment measures were different between phenotypes 2 and 4 only (Figure 10A). Further, flexion-extension range were different between phenotypes 1 and 2 as well as 2 and 4. Considering lumbar movement regarding lateral flexion, phenotype 4 differed statistically from all other phenotypes (Figure 10B). Lastly, all phenotypes for the hands/dowel parallel with the string measure differed statistically except phenotype 4 with 1 and 3 (Figure 10C).

## Discussion

The objective of this study was to identify naturally occurring whole body movement pattern phenotypes related to the performance of exemplar movement screening tasks including the DS, RHS and LHS by using pattern recognition (PCA) and machine learning techniques (GMM). Further, we aimed to contrast how kinematic measures commonly used to score these movements differed between the groups. The statistical results demonstrate that while all kinematic measures commonly used to score the RHS demonstrated a main effect, none of the kinematic measures independently were actually different between all emergent phenotypes (Figure 9). Instead, data demonstrate that kinematic measures commonly used score these screening movements are often different between pairs of phenotypes, but not necessarily able to independently distinguish across all groups (Figures 8–10). This suggests that, if screening continues to be performed using visual appraisal of selected kinematics markers, a hierarchical-based decision tree approach (i.e., first classify on marker A, then sub-classify within those groups based on marker B, etc.) is likely to improve the ability to truly classify and distinguish between groups. Alternatively, contrary to the common practice of analyzing movements based on *a priori* prescribed features, objective data driven analysis can identify and cluster relevant movement phenotypes while considering the system as a whole. Using data driven methods to determine common patterns of movement tasks can reduce the need to use subjective visual appraisal based on *a priori* prescribed features. However, it is also important to note that while this study has generated insights about emergent movement phenotypes during the performance of the DS and hurdle step as exemplar movement screening tasks, the pattern recognition and machine learning techniques applied in this study cannot be applied blindly.

Independent evaluation of discrete kinematics features may not adequately distinguish and separate movement phenotypes. This is particularly evident when analyzing how the phenotypes relate back to the FMS^TM^ scoring criteria, as the means of some scoring criteria were significantly different between phenotypes and others were not. This demonstrates that kinematic measures commonly used to score movements likely shouldn’t be considered independently, and that the interaction between multiple variables might be more revealing. For example, the results for the DS demonstrate significant differences between groups 1 and 2 for two different features (thigh angle and trunk-shank angle difference) and 1 and 3 for three different features (thigh angle, dowel alignment measure and knee-ankle separation ratio). This suggests that the thigh angle or dowel alignment measure could be used to first extract phenotype 1 DS movements (i.e., exceed an appropriate DS thigh angle or dowel alignment threshold), and also highlights a potential redundancy in the DS scoring criteria. However, there were no significant differences present between phenotypes 2 and 3 in any of the measures, which suggests that there are other features that differentiate these groups. It also reveals an important limitation regarding the use of *a priori* prescribed kinematic measure, in that a top-down *a priori* assignment of variables may not actually coincide with kinematic outcomes that do indeed differentiate between groups (Bennett et al., 2017). In this case a sub-ordinate criterion is necessary to distinguish between the movements that remain after screening out phenotype 1 movements by using the DS thigh angle or dowel alignment measure. Highlighting the strengths of pattern recognition and machine learning, these techniques can be applied “bottom-up” to probe for other measures that might better distinguish between DS phenotype 2 and 3. Using our single component reconstructions as a starting point, the frontal view visual representation of the DS phenotypes (Figure 5) shows that foot width was different between phenotypes 1 and 2, and 2 and 3 and has the potential to be a sub-ordinate measure to distinguish between phenotypes. While participants are instructed to place their feet approximately shoulder width apart, some individuals may place their feet wider due to limitations (e.g., morphological). Although this is not a factor that the FMS considers, the use of a wider base may be a result of some functional or behavioral differences that clinicians can explore.

Interpreting results from the LHS and RHS also reveal important evidence underscoring limitations in the use of “top-down” discrete *a priori* measures to score screening movements and further reinforcing the utility of pattern recognition and clustering approaches as “bottom-up” strategies to identify movement phenotypes. In comparison to the FMS^TM^ based kinematic measures used to score the RHS, the clustering revealed that not all features were significant between groups and may be differentially affected in the synergistic control of movement features, further reinforcing the potential of a hierarchical-based approach to screening. For example, the hip-knee, knee-ankle, and hip-ankle alignments were all statistically different between phenotypes 2 and 3, indicating redundancy in the ability of these measures to differentiate, but also in the ability of any one of these measures to be a useful initial measure. However, only the hip-ankle alignment was statistically different between phenotypes 1 and 3, suggesting that this measure could be useful as a sub-ordinate to further refine grouping assignments. Similarly, when considering the lumbar motion related criteria, range of motion about each axis was different between phenotypes 2 and 3, emphasizing redundancy. Nevertheless, the results suggest the ability to differentiate between phenotypes 1 and 2 based on lumbar flexion extension range, and groups 1 and 3 based on the lumbar lateral flexion and lumbar rotation ranges. Like the DS, the use of a bottom-up, data-driven approach has also revealed a factor that might be important, but that is not currently considered: anterior-posterior centre of mass (COM) range of motion (Figure 6). Phenotype 3 (RHS), as an example, elicited a larger range of motion of the COM in the anterior direction, which could not be explained by lumbar angle range of motion, suggesting a possible necessity as an additional movement assessment consideration.

For the LHS, the hip-ankle alignment was the only feature that showed differences between groups for the frontal plane hip-knee-ankle alignment measures. The results demonstrated that phenotype 4 can be differentiated from the other phenotypes based on the lateral lumbar flexion range, but would need subsequent analysis to differentiate further. Whereas, the feature separating phenotypes 1 and 2 and 2 and 4 were due to lumbar lateral flexion range, again supporting a hierarchical approach to screening in the absence of direct data-driven methods. Further, the dowel/hands parallel to string measure elicited differences for phenotype 1 from 2 and 3, as well as phenotype 2 from 3 and 4, thus demonstrating that the kinematic representation of keeping the dowel parallel to the string of FMS^TM^ scoring criteria is a useful tool for differentiating differences in the hierarchy for the LHS. Considering that there were few differences between the frontal plane hurdling leg alignment, perhaps this is a feature that does not demonstrate as much variance as the RHS. Moreover, at this point in the analysis we are not able to explicitly state why the number of optimal clusters differs between the LHS and RHS, but speculate that there may be more variability in the LHS compared to the RHS possibly due to foot dominance. Unfortunately, we did not record foot dominance so we cannot further verify this speculation. Further analysis would be needed to identify the specific kinematic features that further aid to differentiate in the hypothesized hierarchical approach.

This study applied PCA and GMM to a dataset of DS, RHS, and LHS movements as performed by healthy individuals. As a result, the grouping assignments and underlying kinematic difference will likely be different among samples, or perhaps even a larger sample, although the FMS^TM^ has a target population of healthy, active individuals within the general population (Bennett et al., 2017), so our sample may be representative. However, with access to such a larger, representative dataset, this paper provides evidence to support and inform how motion capture, pattern recognition and machine learning can advance movement screening approaches. But, it is also important discuss the assumptions and challenges that emerge when deploying this approach.

One challenge that emerged earlier on in the process of using PCA to identify principal movements was the determination of how many principal movements (PCs) to retain. In this study, PCs that individually explain >5% variance were kept and retained for analysis (Witte et al., 2010), since this method elicited the least number of PCs. However, other common strategies for PC retention include: PCs retained until a trace criterion of 90% of the total variance was retained (Deluzio and Astephen, 2007; Deluzio et al., 2014, p. 322) and PCs retained until a trace criterion of 95% of the total variance was retained (Deluzio et al., 2014, p. 322). However, in other applications, such as optimizing the prediction of a dependent variable, retaining PCs that individually explain >5% variability may not be sufficient (Richter et al., 2014). While retaining a greater number of PCs will include more of the variance within the dataset, when working with clustering, reducing the dimensionality is an important consideration, reinforcing our selection of a PC retention strategy that balance the variance explained with the number of PCs retained.

We chose a GMM as our clustering approach although other types of clustering algorithms may be considered. A GMM was chosen for its advantages of being a distribution-based model. GMM is a soft clustering method based on how probable it is that all data points in the cluster belong to the same distribution. This is contradictory to a centroid-based model with hard clustering (i.e., k-means clustering), where the notion of clustering is based on how close each data point is to the centroid and are assigned to a cluster without considering its variance. While this distinction may not be critical for this paper, it has important implications when clustering for the purpose of movement screening. Considering human variability (within and between), philosophically, it is unlikely that any one individual will absolutely cluster the same way every time. Instead, movements are likely to look more or less like a representative cluster (mean movement), where the GMM can provide an estimate of that likelihood. Such likelihood estimates may inform a hierarchical assessment approach, whereas a mover could be considered not just on the clustered assignment, but also on their likelihood weighting with regards to their assignment to each cluster. This is a concept that requires further contemplation and investigation.

Selecting the optimal number of clusters is also an important consideration, particularly when aiming to quickly screen a wide population of movers, such that they can be appropriately triaged (i.e., identify movers that require targeted training to improve). With the distribution, soft clustering-based method, GMM, the clusters can represent different ellipsoid shapes, overlap or be relatively close to one another which can skew results determined by a method such as a silhouette analysis. Silhouette analysis measures the separability of the clusters based on how close each point in one cluster is to points in the neighboring clusters (Beaudette et al., 2019). As an alternative, the BIC is a criterion for model selection among a finite set of models partly based on the likelihood function. The lower the BIC, the better the model to predict using the data, this model avoids overfitting by penalizing models with big number of clusters (Bishop, 2006, p. 217). Although this may be interpreted as a drawback, if we want to be able to generalize our phenotypes for the purpose of rapid screening or movement-based triage, it is better to penalize large number of clusters. However, if the intent was to support a more personalized diagnoses, an alternate interpretation of the BIC may be required.

### Limitations

Limitations related to the sample size, kinematic trajectories chosen to represent whole-body motion, kinematic variables chosen to represent the scoring criteria and decisions required to apply PCA and GMM likely all have some influence on the results and interpretation of these data. Specific to sample size, the sample size for this study was originally intended for a different research question. However, given that we did not know how many clusters would emerge, we were challenged upon determining the *a priori* sample size. We hope moving forward this study will assist in determining *a priori* sample size. While remaining limitations have been discussed within the main body of the paper, this method nevertheless does show that objective whole-body evaluation can identify phenotypes within a data set. With further research, this method may prove useful and promising in eliminating the subjective assessment of movement screens and improving interrater reliability, or at a minimum, informing on a hierarchy of distinguishable measures that can be used to differentiate movements. It is also important to note that future studies should consider adding their classification code to enable other researchers to use their methods. At this point we are not able to differentiate “good” versus “bad” movers, although our group continues to explore this possibility (Armstrong et al., 2019).

## Conclusion

Overall, pattern recognition and machine learning techniques were able to objectively identify phenotypes within a group of individuals performing the DS, RHS and LHS. Further, when comparing kinematic measures commonly used to score movement between the different phenotype groups, some criteria were indeed different and others were not. In most cases, independent kinematic measures were not able to distinguish between all three/four different emergent phenotypes, and several measures overlapped in their ability to differentiate between phenotype groups. In the absence of objective, data-driven movement assessment, our results suggest that visual-based screening can likely be improved by reducing the number of measures to consider by eliminating independent measures that provide redundant information (i.e., measures that are likely coordinated in their control), and by considering measures using a hierarchical approach (i.e., screen based on measure A, then screen emergent groups based on sub-ordinate measures as necessary). Objective data analysis using whole body movement patterns gives insight into features of the DS and hurdle step that may not be elicited through *a priori* feature selection analysis. Therefore, the results from this study provide important findings to the field that open up a number of future study directions, such as identifying which movement strategy could elicit different injury risk factors to advance injury prediction. Moving toward such objective data driven analysis may further enhance the ability to apply movement screening for the purpose of injury risk identification and mitigation.

## Data Availability Statement

The datasets generated for this study are available on request to the corresponding author.

## Ethics Statement

The study was reviewed and approved the University of Waterloo Office of Research Ethics. Participants for this study provided informed written consent prior to participation.

## Author Contributions

SR, DA, and RG organized the database and completed the allotted analysis. All authors contributed to the design of the study, participated in writing the manuscript and manuscript revision, and read and approved the submitted version.

## Conflict of Interest

The authors declare that the research was conducted in the absence of any commercial or financial relationships that could be construed as a potential conflict of interest.

## Abbreviations

DS: Deep squat
GMM: Gaussian mixture model
LHS: Left hurdle step
PCA: Principal component analysis
PC: Principal component
RHS: Right hurdle step.

## References

[B1] Armstrong D. P. Ross G. B. Graham R. B. Fischer S. L. (2019). Considering movement competency within physical employment standards. *Work* 63 603–613. 10.3233/WOR-192955 31282457

[B2] Beach T. A. C. Frost D. M. Callaghan J. P. (2014). FMSTM scores and low-back loading during lifting – Whole-body movement screening as an ergonomic tool? *Appl. Ergon.* 45 482–489. 10.1016/J.APERGO.2013.06.009 23876984

[B3] Beaudette S. M. Zwambag D. P. Graham R. B. Brown S. H. M. (2019). Discriminating spatiotemporal movement strategies during spine flexion-extension in healthy individuals. *Spine J.* 19 1–12. 10.1016/j.spinee.2019.02.002 30742973

[B4] Bennett H. Davison K. Arnold J. Slattery F. Martin M. Norton K. (2017). Multicomponent musculoskeletal movement assessment tools: a systematic review and critical appraisal of their development and applicability to professional practice. *J. Strength Condit. Res.* 31 2903–2919. 10.1519/JSC.0000000000002058 28614164

[B5] Bennetts C. J. Owings T. M. Erdemir A. Botek G. Cavanagh P. R. (2013). Clustering and classification of regional peak plantar pressures of diabetic feet. *J. Biomech.* 46 19–25. 10.1016/j.jbiomech.2012.09.007 23089457 PMC4538932

[B6] Bishop C. M. (2006). *Pattern Recognition and Machine Learning.* New York, NY: Springer.

[B7] Bock C. Stierli M. Hinton B. Orr R. (2016). The functional movement screen as a predictor of police recruit occupational task performance. *J. Bodywork Mov. Ther.* 20 310–315. 10.1016/J.JBMT.2015.11.006 27210848

[B8] Brandon S. C. E. Graham R. B. Almosnino S. Sadler E. M. Stevenson J. M. Deluzio K. J. (2013). Interpreting principal components in biomechanics: representative extremes and single component reconstruction. *J, Electromyogr. Kinesiol.* 23 1304–1310. 10.1016/j.jelekin.2013.09.010 24209874

[B9] Cohen J. (1988). *Statistical Power Analysis for the Behavioral Sciences*, 2nd Edn Hillsdale, NJ: Lawrence Erlbaum Associates.

[B10] Cook G. Burton L. Hoogenboom B. (2006a). Pre-participation screening: the use of fundamental movements as an assessment of function - part 1. *North Am. J. Sports Phys. Ther.* 1 62–72. PMC2953313 21522216

[B11] Cook G. Burton L. Hoogenboom B. (2006b). Pre-participation screening: the use of fundamental movements as an assessment of function – part 2. *North Am. J. Sports Phys. Ther.* 1 132–139. PMC2953359 21522225

[B12] Deluzio K. J. Astephen J. L. (2007). Biomechanical features of gait waveform data associated with knee osteoarthritis. An application of principal component analysis. *Gait Posture* 25 86–93. 10.1016/j.gaitpost.2006.01.007 16567093

[B13] Deluzio K. J. Harrison A. J. Coffey N. Caldwell G. E. (2014). *Research Methods in Biomechanics.* Champaign, IL: Human Kinetics.

[B14] Federolf P. Reid R. Gilgien M. Haugen P. Smith G. (2014). The application of principal component analysis to quantify technique in sports. *Scand. J. Med. Sci. Sports* 24 491–499. 10.1111/j.1600-0838.2012.01455.x 22436088

[B15] Frost D. M. Beach T. A. C. Mcgill S. M. Callaghan J. P. (2015). The predictive value of general movement tasks in assessing occupational task performance. *Work* 52 11–18. 10.3233/WOR-141902 24962299

[B16] Gilles M. A. Wild P. (2018). Grasping an object at floor-level: is movement strategy a matter of age? *Appl. Ergon.* 70 34–43. 10.1016/j.apergo.2018.02.002 29866323

[B17] Gross D. P. Battié M. C. (2006). Does functional capacity evaluation predict recovery in workers’ compensation claimants with upper extremity disorders? *Occup. Environ. Med.* 63 404–410. 10.1136/oem.2005.020446 16551753 PMC2078106

[B18] Halilaj E. Rajagopal A. Fiterau M. Hicks J. L. Hastie T. J. Delp S. L. (2018). Machine learning in human movement biomechanics: best practices, common pitfalls, and new opportunities. *J. Biomech.* 81 1–11. 10.1016/j.jbiomech.2018.09.009 30279002 PMC6879187

[B19] Hewett T. E. Myer G. D. (2011). The mechanistic connection between the trunk, hip, knee, and anterior cruciate ligament injury. *Exerc. Sport Sci. Rev.* 39 161–166. 10.1097/JES.0b013e3182297439 21799427 PMC4168968

[B20] Holger Hoffmann (2020). *Violin Plot.* Available at: https://www.mathworks.com/matlabcentral/fileexchange/45134-violin-plot (accessed March 25, 2020).

[B21] Isernhagen S. J. (1992). Functional capacity evaluation: rationale, procedure, utility of the kinesiophysical approach. *J. Occup. Rehabil.* 2 157–168. 10.1007/BF01077187 24243032

[B22] Kiesel K. Plisky P. J. Voight M. L. (2007). Can serious injury in professional football be predicted by a preseason functional movement screen? *North Am. J. Sports Phys. Ther.* 2 147–158. 21522210 PMC2953296

[B23] Kritz M. Cronin J. Hume P. (2009). The bodyweight squat: a movement screen for the squat pattern. *Strength Condit. J.* 31 76–85. 10.1519/SSC.0b013e318195eb2f

[B24] Leardini A. Chiari L. Della Croce U. Cappozzo A. (2005). Human movement analysis using stereophotogrammetry: part 3. Soft tissue artifact assessment and compensation. *Gait Posture* 21 212–225. 10.1016/j.gaitpost.2004.05.002 15639400

[B25] Lisman P. O’Connor F. G. Deuster P. A. Knapik J. J. (2013). Functional movement screen and aerobic fitness predict injuries in military training. *Med. Sci. Sports Exerc.* 45 636–643. 10.1249/MSS.0b013e31827a1c4c 23190584

[B26] McCunn R. Aus der Fünten K. Fullagar H. H. K. McKeown I. Meyer T. (2016). Reliability and association with injury of movement screens: a critical review. *Sports Med.* 46 763–781. 10.1007/s40279-015-0453-1 26721517

[B27] McGill S. Frost D. Lam T. Finlay T. Darby K. Cannon J. (2015). Can fitness and movement quality prevent back injury in elite task force police officers? A 5-year longitudinal study. *Ergonomics* 58 1682–1689. 10.1080/00140139.2015.1035760 25952105

[B28] Mottram S. Comerford M. (2008). A new perspective on risk assessment. *Phys. Ther. Sport* 9 40–51. 10.1016/J.PTSP.2007.11.003 19083703

[B29] Moudy S. Richter C. Strike S. (2018). Landmark registering waveform data improves the ability to predict performance measures. *J. Biomech.* 78 109–117. 10.1016/j.jbiomech.2018.07.027 30126719

[B30] O’Connor F. G. Deuster P. A. Davis J. Pappas C. G. Knapik J. J. (2011). Functional movement screening: predicting injuries in officer candidates. *Med. Sci. Sports Exerc.* 43 2224–2230. 10.1249/MSS.0b013e318223522d 21606876

[B31] Okada T. Huxel K. C. Nesser T. W. (2011). Relationship between core stability, functional movement, and performance. *J. Strength Condit. Res.* 25 252–261. 10.1519/JSC.0b013e3181b22b3e 20179652

[B32] Ortiz A. Rosario-Canales M. Rodríguez A. Seda A. Figueroa C. Venegas-Ríos H. L. (2016). Reliability and concurrent validity between two-dimensional and three-dimensional evaluations of knee valgus during drop jumps. *Open Access J. Sports Med.* 7 65–73. 10.2147/OAJSM.S100242 27313480 PMC4890697

[B33] Parchmann C. J. McBride J. M. (2011). Relationship between functional movement screen and athletic performance. *Strength Condit.* 25 3378–3384. 10.1519/JSC.0b013e318238e916 21964425

[B34] Powers C. M. (2010). The influence of abnormal hip mechanics on knee injury: a biomechanical perspective. *J. Orthop. Sports Phys. Ther.* 40 42–51. 10.2519/jospt.2010.3337 20118526

[B35] Richter C. McGuinness K. O’Connor N. E. Moran K. (2014). The variance needed to accurately describe jump height from vertical ground reaction force data. *J. Appl. Biomech.* 30 732–736. 10.1123/jab.2013-0313 25010220

[B36] Robertson G. Caldwell G. Hamill J. Kamen G. Whittlesey S. (2013). *Research Methods in Biomechanics, 2E.* Champaign, IL: Human Kinetics.

[B37] Roche N. Pradon D. Cosson J. Robertson J. Marchiori C. Zory R. (2014). Categorization of gait patterns in adults with cerebral palsy: a clustering approach. *Gait Posture* 39 235–240. 10.1016/j.gaitpost.2013.07.110 23948331

[B38] Ross G. B. Dowling B. R. Troje N. F. Fischer S. L. Graham R. B. (2018). Objectively differentiating movement patterns between elite and novice Athletes. *Med. Sci. Sport Exerc.* 50 1457–1464. 10.1249/MSS.0000000000001571 29420437

[B39] Sawacha Z. Guarneri G. Avogaro A. Cobelli C. (2010). A new classification of diabetic gait pattern based on cluster analysis of biomechanical data. *J. Diabetes Sci. Technol.* 4 1127–1138. 10.1177/193229681000400511 20920432 PMC2956820

[B40] Schneiders A. G. Davidsson A. Hörman E. Sullivan S. J. (2011). Functional movement screen normative values in a young, active population. *Int. J. Sports Phys. Ther.* 6 75–82. 21713227 PMC3109893

[B41] Shultz R. Anderson S. C. Matheson G. O. Marcello B. Besier T. (2013). Test-retest and interrater reliability of the functional movement screen. *J. Athletic Train.* 48 331–336. 10.4085/1062-6050-48.2.11 23675792 PMC3655746

[B42] Sinden K. E. McGillivary T. L. Chapman E. Fischer S. L. (2017). Survey of kinesiologists’ functional capacity evaluation practice in Canada. *Work* 56 571–580. 10.3233/WOR-172519 28339418

[B43] Srinivasan D. Mathiassen S. E. (2012). Motor variability in occupational health and performance. *Clin. Biomech.* 27 979–993. 10.1016/j.clinbiomech.2012.08.007 22954427

[B44] Toro B. Nester C. J. Farren P. C. (2007). Cluster analysis for the extraction of sagittal gait patterns in children with cerebral palsy. *Gait Posture* 25 157–165. 10.1016/j.gaitpost.2006.02.004 16647260

[B45] Troje N. F. (2002). Decomposing biological motion: a framework for analysis and synthesis of human gait patterns. *J. Vis.* 2 371–387. 10.1167/2.5.2 12678652

[B46] Watelain E. Barbier F. Allard P. Thevenon A. Angué J. C. (2000). Gait pattern classification of healthy elderly men based on biomechanical data. *Arch. Phys. Med. Rehabil.* 81 579–586. 10.1016/s0003-9993(00)90038-8 10807095

[B47] Winter D. A. (2009). *Biomechanics and Motor Control of Human Movement.* Hoboken, NJ: John Wiley & Sons.

[B48] Witte K. Ganter N. Baumgart C. Peham C. (2010). Applying a principal component analysis to movement coordination in sport. *Math. Comput. Model. Dyn. Syst.* 16 477–488. 10.1080/13873954.2010.507079

[B49] Wrigley A. T. Albert W. J. Deluzio K. J. Stevenson J. M. (2005). Differentiating lifting technique between those who develop low back pain and those who do not. *Clin. Biomech.* 20 254–263. 10.1016/j.clinbiomech.2004.11.008 15698697

[B50] Wu G. Siegler S. Allard P. Kirtley C. Leardini A. Rosenbaum D. (2002). ISB recommendation on definitions of joint coordinate system of various joints for the reporting of human joint motion—part I: ankle, hip, and spine. *J. Biomech.* 35 543–548. 10.1016/s0021-9290(01)00222-6 11934426

[B51] Wu G. van der Helm F. C. T. DirkJan Veeger H. E. J. Makhsous M. Van Roy P. Anglin C. (2005). ISB recommendation on definitions of joint coordinate systems of various joints for the reporting of human joint motion—Part II: shoulder, elbow, wrist and hand. *J. Biomech.* 38 981–992. 10.1016/j.jbiomech.2004.05.042 15844264

[B52] Zazulak B. Cholewicki J. Reeves N. P. (2008). Neuromuscular control of trunk stability: clinical implications for sports injury prevention. *J. Am. Acad. Orthop. Surg.* 16 497–505. 10.5435/00124635-200808000-00011 18768707

